# Hypertension and brain damage: evidence from rodent models

**DOI:** 10.1186/s42826-026-00270-0

**Published:** 2026-03-24

**Authors:** Marta Sofia Scenna, Eleonora Maceroni, Annamaria Cimini, Vanessa Castelli, Michele d’Angelo

**Affiliations:** 1 Department of Life, Health and Environmental Sciences, University of L’Aquila, 67100 L’Aquila, Italy; 2 Sbarro Institute for Cancer Research and Molecular Medicine, Department of Biology, Temple University, Philadelphia, PA USA

**Keywords:** Chronic hypertension, Brain, Rats, BBB, Small vessel disease

## Abstract

Hypertension is a prevalent condition that significantly raises the incidence of cerebrovascular and cognitive disorders. This review focuses on the factors most closely linked to stroke, cognitive impairment, and Alzheimer’s disease. Research into pathophysiology and treatment of hypertensive brain damage has greatly benefited from rodent models, which have been crucial in uncovering the underlying mechanisms and developing effective therapeutic strategies. Rodent models, particularly spontaneously hypertensive rats (SHR) and stroke-prone SHR (SHR-SP), have been essential in elucidating the pathophysiological mechanisms connecting hypertension to brain damage. These models exhibit structural and functional cerebrovascular alterations, including blood-brain barrier disruption, microvascular rarefaction, and neuroinflammation. Interventions targeting the renin-angiotensin system have shown promise in mitigating these adverse effects. This review synthesizes current findings from rodent studies, underscoring the pivotal impact of hypertension in brain pathology and the potential therapeutic benefits of antihypertensive treatments.

## Background

The diagnosis of hypertension is made when the systolic blood pressure reading is greater than or equal to 130 mmHg and/or the diastolic blood pressure reading is greater than or equal to 90 mmHg on two different days [1]. Hypertension affects 1.13 billion people worldwide, representing a significant proportion of the global population. Among cardiovascular diseases, high blood pressure is a leading risk factor for lethal complications, particularly in older individuals, resulting in 9.4 million deaths annually [2].

In individuals with no underlying health conditions, blood pressure (BP) levels stay inside the normal range due to a delicate equilibrium between factors that might raise BP and those that regulate it, serving as restorative system [2]. In hypertension, these mechanisms are disrupted, leading to various damages, by affecting the brain in the first instance [3].

Hypertension drives inward remodeling of cerebral arterioles and attenuates blood delivery to cerebral capillaries, compromising microvascular perfusion [4]. This decline contributes to brain damage by reducing the intake of nutrients and contributing to neurovascular disruption [5]. Moreover, the loss of cerebral autoregulation due to hypertension is correlated with an enhanced transmission of pressure to cerebral capillaries, which participates in vascular remodeling, blood-brain barrier (BBB) dysfunction [6], and small vessel disease [7].

High blood pressure induces extensive cerebrovascular remodeling and dysfunction, which are major contributors to stroke and vascular cognitive decline [5, 7]. Furthermore, hypertension-related vascular dysfunction is increasingly recognized as a key factor in Alzheimer’s disease pathogenesis [5].

Understanding the intricate correlation between hypertension and brain damage is crucial for developing effective therapeutic strategies. Rodent models, particularly SHR and SHR-SP, have provided valuable insights into the mechanisms underlying hypertension-induced brain pathology. These models mimic many aspects of human hypertension and its neurological consequences, making them indispensable tools for research. This paper aims to review the current evidence from rodent studies, highlighting the morphological and functional changes in the brain correlated with chronic hypertension and exploring potential interventions to mitigate these effects (Table 1).

**Table 1:** Structural and functional changes in the brain associated with chronic hypertension

**Structural changes**	Increased intraluminal pressure in the vessels [8].	Compressive force on the arterial wall [8].	Inward remodeling [4].	Hypertrophy [9].	Decrease in cerebral blood flow [10].	Lower cerebrovascular reserve [11].
**CBF autoregulation**	Loss of cerebral autoregulation [6].	Increased transmission of pressure to cerebral capillaries [12].	Leakage of BBB [6].	Cerebral edema [12].	Inflammation [12].	Neuron degeneration [12].
**Vascular Remodeling and BBB dysfunction**	ROS production [8].	Decreased pericyte coverage [6].	Loss of BBB integrity [6].	Collagen buildup in the perivascular space [6].	Tight junction remodeling and increased transcytosis [13].	Permeability dysfunction [4].
**CSVD**	Subcortical white matter damage [4].	Lacunar infarcts and microinfarcts [7].	Microhemorrhages [14].	Arteriolosclerosis [7].	Impaired cerebral blood flow [10].	Failure in amyloid clearance [15].
**Disruption of neurovascular function**	Loss and narrowing of small cerebral vessels [7].	Endothelial and BBB Dysfunction [7].	ROS production by perivascular macrophages [16].	Compromised maintenance of CBF [17].	Early functional deficits that precede neuronal damage [18].	Suppressed neurovascular coupling [19].

## Main text

### Rodent models of hypertension-associated brain damage

The study of complex and multifactorial pathologies cannot be separated from the use of an in vivo model. In the context of hypertension, mouse models related to rare conditions such as, for example, nephrovascular hypertension or Page’s kidney were initially developed. Focusing on primary hypertension, there have been more difficulties in developing mouse models, especially because the disease mechanisms in humans are complex and multifaceted [20]. Mostly, rats with hypertension used as an in vivo model are derived from strains of Wistar or Sprague-Dawley that have been selected for traits related to high blood pressure. Below, an overview of the most used strains.

#### Spontaneously Hypertensive Rat (SHR)

From the mating between a male Wistar with marked traits of high blood pressure and a female with moderately high blood pressure values, the SHR strain was originated in Kyoto. Subsequently, animals with systolic blood pressure values above 150 mmHg were selected from sibling mating. It was only at the end of the 1960s, at the National Institutes of Health (United States), that they successfully generated the strain that develops hypertension in adults spontaneously. Although the tendency to manifest stroke is a typical characteristic of Stroke-Prone SHRs, it is interesting to note that even in this model, there is the possibility of it happening. In addition, there are several areas of study in which this animal has been used, including studies of vascular and renal function, gene expression related to hypertension, and evaluation of therapeutic approaches [21].

A work published in 2021 characterized the phenotypic aspects of this model. It was found that in the presence of a standard diet, at six months of age, the systolic blood pressure of SHRs was significantly higher than in Wistar Kyoto (WKY) rat controls. The WKY rat serves as the normotensive control for SHR and SHR-SP models. It shares genetic background but does not develop hypertension, making it essential for distinguishing BP-dependent effects [22]. Adding salt to the diet resulted in a further increase in blood pressure. In addition to abnormalities related to blood pressure levels, problems in lipid metabolism have been described [21].

#### Stroke-prone SHR (SHR-SP)

Starting with the SHR model, the SHR-SP model was generated. Specifically, in the early 1970s, Yamori and colleagues selected a subpopulation that was particularly prone to stroke from SHR rats. The model was then stabilized after 24 generations of selective couplings [23]. Of particular interest is the fact that these animals are not only capable of developing strokes spontaneously but have stroke-related genetic characteristics like those of humans. This makes SHR-SP in vivo model ideal for studying molecular mechanisms underlying the disease, but also for investigating the predictability by genetic analysis [24].

SHR-SP rats immediately have abnormal blood pressure, which, in the absence of treatment, does not improve until the animal dies. Over time, several studies have confirmed similarities with human CSVD, including alterations in cerebral vascular function, compromised integrity of the BBB, abnormalities of micro vessels, leakage of erythrocytes from the parenchyma, determination of punctiform hemorrhages, and sometimes loss of myelin [25]. Interestingly, compared to SHR mice, they show an alteration in the genes that control blood pressure earlier, such as Agt, Agtrap, Ephx2, and Uts2 genes [26].

#### Dahl Salt-Sensitive Rat (Dahl/SS)

In the study of hypertension, a particularly well-known model is represented by the Dahl Salt-Sensitive Rats (Dahl/SS). This model was defined for the first time in the 60s by the union of Sprague-Dawley rats sensitive and non-sensitive to salt. This strain can develop profound hypertension when fed a diet high in salt (2–8%). In addition, over time, real decompensation occurs starting from cardiac hypertrophy [27]. In addition to high blood pressure, these animals tend to develop extensive damage to kidney levels that has marked similarities to the kidney damage seen in patients with hypertension-related chronic kidney disease [28].

In the study of the mechanisms of action, research in 2024 showed that in these animals, a significant intake of sodium favored an increase in plasma concentrations of PCSK9 through SREBP2 activation at both transcription and translation levels. As a result, this activation led to a decrease in hepatic LDL receptors (LDLRs) and the removal of LDL cholesterol from circulation, contributing to the onset of hypercholesterolemia [29]. Among the characteristics that this model shows in common with human hypertension, are abnormal plasma renin activity, lower levels of aldosterone in the circulation, increased vascular resistance, impaired pressure-natriuresis and decreased venous compliance [30].

#### Blood Pressure High (BPH/2J) mice

In the early 1970s, Schlager and collaborators created a mouse model of elevated blood pressure known as BPH/2 [31]. This mouse line was obtained through a selective breeding program, aimed at progressively increasing systolic blood pressure. This strain was developed by using a selection based on pressure values determined by measurements made on the tail using the cuff system [32]. From a phenotypic point of view, compared to BPN/3 reference controls, an overactivation of the sympathetic nervous system is observed, which correlates with constriction of the vessels [33]. In addition, increased adrenal gland activity is observed, thus increased levels of adrenaline and noradrenaline [34]. It is interesting to note that in these animals, high levels of c-fos are observed in the amygdala, especially in the medial amygdaloid nucleus (MeAm) and that lesions in this region lead to lowering BP.

In this model, a large contribution to hypertension is due to the sympathetic nervous system; in fact, the ganglion block can regulate hypertension, leading to levels similar to those of control [35]. Other studies have shown that there may be a contribution due to GABAergic inhibition, as a reduced transcription of the δ, α4, and β2 subunits of the GABA A receptor is observed in the hypothalamus, key features for the tonic inhibition of neurons. Allopregnanolone acts as an allosteric modulator of these receptors and, by increasing their expression in the amygdala and hypothalamus, attenuates hypertension and sympathetic activation. The data collected so far indicates a dysfunction of the GABAergic system in these brain areas that causes excessive neuronal activation and supports sympathetic-mediated hypertension, making BPH/2 a valid model for the study of neurogenic hypertension [36]. Orexin is a neuropeptide that has relevance in blood pressure control, heart rate, and sympathetic activity [37]. In BPH/2 mice, orexin is implicated in hypertension as they show more than double mRNA expression compared to controls [38]. By administering an Orhexin receptor antagonist, researchers documented a marked reduction in BP at night due to both the absence of motor movement but also to modulation of sympathetic activity [39].

This model has great relevance for human hypertension as it has made it possible to highlight that hypertension can be driven at the brain level, not only by hormones or blood vessels. In particular, the hyperactivated amygdala-hypothalamic pathway triggers the activity of the sympathetic nervous system. This indicates that modulation at the level of these brain areas could be a strategy to treat some forms of hypertension in humans as well [40–42].

### Structural changes in hypertension

Chronic hypertension leads to morphological changes in the cerebral arteries and arterioles, which has been detected in animal models of high blood pressure, including the SHR [17]. In chronic hypertension, increased intraluminal pressure in the vessels exerts a compressive force on the arterial wall, resulting in elevated wall tension and stress. A reduction of the radius and increment of the wall thickness is observed in blood vessels to preserve normal wall tension and stress [8]. In cerebral circulation, chronic hypertension induces substantial inward remodeling of arterioles in rat models, particularly in SHR and SHR-SP. Bastrup and Jepps employed DIA-MS proteomic analysis on isolated cerebral arteries from 12-week-old SHR and WKY controls, revealing early signs of vascular hypertrophy and imbalanced expression of angiogenic and extracellular matrix proteins. These molecular alterations were paralleled by morphological changes, such as increased media-to-lumen ratio in basilar arteries, assessed through histological staining [9]. Similarly, studies on 28 week-old SHR-SP, explicitly confirmed to be free from stroke lesions at the time of analysis, demonstrated through pressure myography and morphometric analysis a significant increased vessel walls and a reduction in lumen size, with most of the narrowing attributable to structural rearrangement rather than wall thickening alone [43].There has been extensive research into the mechanisms underlying hypertrophy and inward remodeling. Elevated levels of angiotensin II (Ang II) in hypertension, more than hypertension itself, is thought to be the main cause of arterial and cerebral inner rearrangement. In SHR-SP, internal remodeling was observed to be inhibited by the treatment with the ACE inhibitor perindopril [44]. Moreover, infusion of Ang II for 28 days in healthy mice resulted in internal remodeling in cerebral arterioles, these changes were observed even at moderate increases in blood pressure and persisted after its normalization [45]. Interestingly, high-dose Ang II infusion into transgenic knockout mice demonstrated that caveolin-1-mediated activation was involved in remodeling of cerebral arterioles [46]. Numerous studies have demonstrated that stiffening can decrease cerebral blood flow (CBF) [10], which can lead to lower cerebrovascular reserve, particularly among older individuals [11]. These adverse microvascular effects can be sensitive predictors of the development of subsequently occurring white matter lesions and impaired cognitive function highlighting that arterial stiffening plays a role in these processes. A recent in vivo study using SHR showed that leptomeningeal anastomoses (LMAs), exhibit increased basal vasoconstrictive tone compared to WKY. This elevated tone persisted during ischemia and reperfusion, limiting collateral flow and contributing to reduced perfusion of the ischemic penumbra. Notably, even when systemic blood pressure was pharmacologically increased via phenylephrine, LMAs in SHR remained constricted [47]. This underscores the importance of comprehending hypertensive vascular damage in stroke prognosis, as does chronic hypoperfusion, which can result in further stroke and cognitive decline [48]. Similar vascular remodeling has also been observed in the Dahl salt-sensitive (Dahl/SS) rat model. Central arterial stiffness was found to drive hippocampal hypoperfusion and decreased N-acetyl aspartate levels, which is a marker of neuronal loss, indicating early neurovascular dysfunction [49].

### Cerebral blood flow autoregulation

Due to the restricted energy reserves, the brain relies on a significant transfer of oxygen and glucose throughout the bloodstream to function at its optimal capacity. For this purpose, it regulates cerebral blood flow in an extremely efficient manner, ensuring that perfusion pressure fluctuations are effectively managed. It has been demonstrated that blood flow in the brain can be preserved at a relatively constant level when mean arterial pressure (MAP) is within physiological autoregulatory limits. However, when arterial pressure falls below or rises above these limits, autoregulation is impaired [50].

Naessens et al. assessed the ability of cerebral arteries in untreated SHR to respond to incremental pressure increases using ex vivo pressure myography. The vessels showed a blunted contractile behavior across a range of physiological pressures (60–140 mmHg), indicating a dysfunctional myogenic mechanism. This impaired response may comprise the maintenance of CBF under varying perfusion conditions, limiting autoregulatory efficiency in this hypertensive model [17]. If BP is not maintained at autoregulatory levels, there is a significant danger of cerebral injury. For instance, if cerebral perfusion pressure declines under the lower threshold of autoregulatory control, brain ischemia will occur, which can lead to permanent damage [51]. Furthermore, the loss of cerebral autoregulation is related with an increased transmission of pressure to cerebral capillaries, potentially leading to disruption of the blood-brain barrier, cerebral edema, inflammation, and neuron degeneration, which are commonly observed in subjects with vascular cognitive impairment (VCI) [12].

### Vascular remodeling and BBB dysfunction

Chronic hypertension leads to changes in blood vessels known as vascular remodeling, which is believed to contribute to damage in various organs. One common manifestation of this in the brain is microcirculatory dysfunction, leading to reduced vasoreactivity, microbleeds, and infarcts (areas of dying tissue due to insufficient blood supply) in the brain tissue over time. Research from *post-mortem* studies has shown that vascular remodeling occurs due to an accumulation of extracellular matrix, primarily affecting small blood vessels like arterioles, venules, and capillaries. In a widely used animal model of chronic hypertension, using SHRs, research has shown a decrease in the number of capillaries and leakage of the BBB as a result of prolonged high blood pressure [6]. Furthermore, these trials showed that hypertension is linked to an increase in the thickness of the capillary wall and a deposition of collagen in the capillary basement membrane, as well as brain pericyte damage. In a recent study published in 2021 [6], it was demonstrated that high blood pressure induces collagen accumulation around microvessels, leading to decreased pericyte coverage and loss of BBB integrity. Surprisingly, the primary contributors to the perivascular collagen accumulation were identified as pericytes. These results suggest that alterations in the BBB occur prior to the reduction in cerebral microvessel density and the onset of cognitive decline in individuals with chronic hypertension. Notably, BBB dysfunction has been proposed as an early indicator of cognitive dysfunction in Alzheimer’s disease. Furthermore, the chronic hypertension model showed a higher buildup of extracellular matrix surrounding the impacted microvessels. This suggests that pericytes could be reacting by boosting collagen production in response to the elevated levels of inflammatory substances, such as TGFβ1, found in the blood of SHRs. The increased collagen deposition might then hinder pericyte-endothelium interactions, ultimately resulting in BBB dysfunction [6].

The BBB is designed to control trafficking of molecules inside and outside of the brain [52]. The tight junctions (TJ) that connect adjacent endothelial brain cells, avoid the paracellular passage of molecules. These cells also exhibit limited vesicular transport and fine regulation of the exchange of molecules across blood and brain due to the expression of specific luminal transporters like fatty acid transport protein 1 (FATP-1) [13], and the MFSD2A [53]. The development and preservation of the BBB depend on the interplay amongst endothelial cells, as well as on the interaction of these cells with other constituents of the neurovascular unit, such as pericytes, perivascular cells, and astrocytes [54].

It is well documented that alterations of the BBB may be due to hypertension. Different studies have pointed out that vascular wall damage, especially in venules, is associated to the acute elevation of BP [55]. Several investigations have studied the effects of agents implicated in human high blood pressure, like Ang II, to endothelial cell permeability indices [56]. Using chronic Ang II-dependent and genetic models of hypertension, researchers analyzed the cellular mechanisms that result in increased BBB permeability. They found that prolonged elevation of blood pressure caused by Ang II results in TJ rearrangement and enhanced transcytosis. In permeability dysfunction, Ang II type 1 receptors (AT1Rs) on endothelial cells play a crucial role in its initiation. However, it is AT1Rs in perivascular macrophages (PVMs) that are essential for the complete manifestation of the malfunction, facilitated by the free radical-producing enzyme Nox2. This highlights the importance of brain endothelial/perivascular innate immune cell communication in the establishment of BBB dysfunction [4].

By analyzing the transit of horseradish peroxidase (HRP) in the blood in different models of hypertensive rats, it was confirmed a state of chronic hypertension correlated with BBB damage [22]. Specifically, SHR, SHR with stroke (SHR-SP) and Wistar Kyoto rats (WKY) at three months of age were used and injected with HRP. In SHR and SHR-SP, loss of HRP was observed in the vessels along the hippocampal fissure [57]. The ultrastructural examination of SHR-SP showed that HRP was found in vesicular structures within the cytoplasm of endothelial cells, in the basal lamina, and extracellular space. Additionally, signs of collagen accumulation indicating increased vascular permeability were evident in the basal lamina of the vessel wall. These observations suggested that chronic hypertension leads to significant damage to the BBB.

### Cerebral small vessel disease

High blood pressure is the primary driver of microvascular abnormalities leading to subcortical white matter damage, known as cerebral small vessel disease (CSVD), which is a usual contributor to cognitive impairment [58].

CSVD is strongly correlated with age and is a prominent cause of lacunar stroke and contributes significantly to vascular impairment of the brain and its subsequent effects on cognitive function and dementia [7]. Areas of the brain most affected by CSVD include the subcortical white and periventricular white matter as well as the deep gray nuclei [7].

Neuropathological consequences include abnormalities in white matter (visible as areas of high intensity on T2-weighted MRI), small ischemic areas (also known as *lacunae* or microinfarcts), and, less frequently, subcortical microhemorrhages [14]. The most common manifestation of CSVD is arteriolosclerosis, which is described as fibrotic thickening at the level of small penetrating arteries (up to 300 microns in outer diameter can be reached). There are also other CSVD forms (arterioma of small arteries and lipohyalinosis) although they are reported more rarely. Additional lesions embrace cerebral amyloid angiopathy and venous collagenosis [7].

CSVD can be divided into amyloid and non-amyloid forms [59]. A common form of amyloid CSVD is cerebral amyloid angiopathy (CAA), described by the presence of amyloid β-portin (Aβ) fibrils in the brain. It is a important complication in the most patients with Alzheimer’s disease and appears sporadically in > 50% of individuals older than 80 years [41]. Two types of CAA can be distinguished: in type 1 CAA, fibrils form mainly in the microvessels and capillaries of the brain, whereas in type 2 CAA small intracortical arteries and arterioles and superficial meningeal/pial vessels are involved [41].

Non-amyloid forms of CSVD encompass various lifestyle-related conditions including high blood pressure [60] and diabetes [61]. As analyzed above, hypertension causes damage in the integrity of the BBB. In addition, endothelial malfunction is another important cause of cerebral hemorrhage, WM damage, VCI and vascular cognitive impairment and dementia (VCID) [5]. Hypertension is diagnosed in approximately 70% of individuals above 65 years old and is present in 80% of patients with CSVD, making it a clear risk factor for VCID [62].

Studies on SHR-SP have elucidated that hypertension can result in a failure of Aβ clearance, and the resulting CVSD can lead to the determination of CAA [15]. Early vascular alteration, including BBB disruption, vessel wall injury, and thrombotic events, were demonstrated using in vivo two photon microscopy, CBF measurements, and ex vivo immunohistochemistry (Aβ, IgG, lectins). These alterations were temporally antecedent to amyloid deposition, supporting the concept that non-amyloid CSVD provides a pathological substrate for secondary vascular amyloidosis. Although the two condition differ in origin, their overlap in the SHR-SP model supports the idea of a continuum in small vessel pathology, where hypertension-induced damage evolves toward secondary ischemic injury and amyloid-related vascular degeneration [15].

### Disruption of neurovascular function

The balance of the brain’s interior environment is upheld through the coordinated efforts of different cells type such as neurons, astrocytes and vascular cells, which operate together as a one functional element (vascular unit) [63]. The increase in synaptic activity leads to an elevation of CBF in the activation zone [64]. This mechanism is known as neurovascular coupling and is the result of the interaction between cells of the neurovascular unit. The precise mechanical basis of the vascular reaction remains unclear. However, it is currently evident that neurons, astrocytes, endothelial cells, smooth vascular muscle cells, and pericytes collaborate in this manner via diffusible factors such as NO, prostaglandins, adenosine, ions, etc. Additionally, they do so through the activation of ion channels and specific signaling pathways [64].

The cause of relaxation, mediated through intramural vascular signaling, is due to activation of neurons leading to vascular changes first in the capillary endothelium and then, by transmission, to the upstream arterioles. In order to induce an improve in flow in the interested area, the larger vessels in the brain are involved thanks to retrograde propagation [65].

The role of vascular endothelium is essential in preserving the health of neurons, glia, and oligodendrocytes. Hypertension, however, has been shown to reduce CBF at rest, which in turn suppresses neurovascular coupling. Nevertheless, latest research has observed both localized and overall decreases in resting CBF among hypertensive patients, a finding that challenges the earlier observations. In patients who are cognitively normal but hypertensive, without other signs of brain abnormalities, these localized reductions in CBF indicate that such changes might precede the onset of brain pathology [19]. There are two potential explanations describing these reductions although the fundamental mechanisms remain unclear: decreased metabolism in the brain or a direct effect of hypertension on the cerebral vascular system [66].

Recent studies have shown that neurovascular coupling to whisker stimulation is compromised in genetic models of persistent hypertension, such as BPH/2J [67] and SHR. Notably, this dysfunction emerged even in the absence of changes in neuronal viability markers, suggesting that vascular dysregulation precedes neuronal damage. Reduced spatial activation in BOLD imaging points to an early mechanistic link between chronic hypertension and cognitive impairment [18].

The subfornilacular organ (SFO), one of the circumventricular organs, is the target of prolonged infusion of Ang II. Secretion of vasopressin and stimulation of endothelin-1 production in cerebral arterioles results in stimulation of hypothalamic pathways [68]. Activation of the central tract SFO is essential for neurovascular impairment caused by Ang II hypertension at slow pressors.

An especially important role is played by oxidative stress, which seems to be implicated in neurovascular impairment in models of Ang II Hypertension. Recently, several studies have shown that perivascular macrophages (PVMs) are important producers of reactive oxygen species (ROS) and their role in mediating the neurovascular dysfunction [69]. Given their myeloid origin, PVMs exhibit functional AT1R and, via Nox2, are able to produce massive amounts of ROS [16]. This topic holds substantial importance as cerebrovascular impairment in slow-pressor Ang II hypertension is primarily caused by vascular oxidative stress, which stems from a Nox2-containing NADPH oxidase [68, 70].

In BPH mice it was seen that fewer PVMs correlated with improved cognitive activity [67]. These data conclude that PVM are important producers of ROS responsible for the critical effects on cognitive function and neurovascularization related to hypertension. Unlike cerebral endothelium, arteriolar smooth muscle does not express AT1R and Nox2, and thus it is possible to suggest that these cells contribute to the ROS production observed in Ang II hypertension. However, their contribution is presumed to be less pronounced considering data obtained from single-cell RNA sequencing indicating limited expression of Nox2 in endothelial cells in comparison with microglia and PVM. In addition, the finding that removal or genetic mutation of PVM results in complete abolition of vascular ROS production [67] indicates that both endothelium and vascular smooth muscle do not play a role in radical production. The fact that, in the hypertensive syndrome due to acute administration of angiotensin II, PVMs do not take part in the process that causes the neurovascular alterations, implies a role the endothelium in ROS production.

Recent longitudinal and multimodal neuroimaging studies have reinforced the view that chronic hypertension compromises neurovascular coupling in response to whisker stimulation in SHR rats.

It is noteworthy that, by six months of age, SHR exhibit a significant reduction in the cortical hemodynamic response, even though neuronal metabolism remains intact [18]. Furthermore, other studies used combined optical and spectroscopic imaging and demonstrated that antihypertensive treatment normalizes resting perfusion but fails to rescue stimulus-evoked blood flow, pointing to persistent vascular dysfunction [71]. Moreover, MRI assessment describe a shift from early transient hyperperfusion to later hypoperfusion and coupling deficits, correlating with microvascular rarefaction and neural cell loss [72].

While extensive data demonstrate impaired whisker-evoked neurovascular coupling in SHR models, functional studies in SHR-SP and Dahl SS rat are lacking in recent literature. This gap may be attributed to the early onset of cerebrovascular lesions in SHR-SP, which complicate functional assessments, and the lack of standardized protocols for Dahl SS rats, whose pathological features vary significantly depending on diet and genetic strain.

See Table 2 for a summary of evidence from rodent models.

**Table 2:** Evidence from rodent models

Model	Rodent model	Evidence	Origin of Damage	References
**Structural Changes**	SHR	Structural remodeling of cerebral arteries and arterioles	Primary hypertension	[17]
		Vascular hypertrophy ↑ media-to-lumen ratio		[9]
		↓ CBF and cerebrovascular reserve due to arterial stiffening		[10]
		↑ vasoconstrictive tone of LMAs persistent during stroke and reperfusion	Secondary ischemic injury	[47]
		Chronic hypoperfusion worsens stroke outcome and promotes cognitive decline		[48]
	SHR-SP	Inward remodeling of cerebral arterioles	Primary hypertension	[43]
		The use of an ACE inhibitor inhibited internal remodeling		[44]
	Transgenic knockout mice	Caveolin-1- mediated activation was involved in remodeling	Primary hypertension	[46]
	Dahl S/S	Central arterial stifness→hippocampal hypoperfusion ↓ N-acetyl aspartate levels	Primary hypertension	[49]
**CBF autoregulation**	SHR	Arteries may be unable to constrict under the prevailing pressures	Primary hypertension	[17]
**Vascular remodeling and BBB dysfunction**	SHR	Inflammatory cytokine-induced collagen production (e.g. TGF-1β) by pericytes → impaired pericyte-endothelial contact and BBB breakdown	Primary hypertension	[6]
		HRP leakage in hippocampus Collagen deposition in basal lamina Ultrasctructural endothelial vescicles	Primary hypertension	[57]
	SHR-SP	HRP leakage in hippocampus Collagen deposition in basal lamina Ultrasctructural endothelial vescicles	Primary hypertension	[57]
	Genetic and Ang II dependent models	Ang II-induced high BP leads to TJ altering and increased transcytosis	Primary hypertension	[4]
**Small Vessel Disease**	SHR-SP	Impaired Aβ clearance	Primary hypertension	[15]
		Early CSVD favors CAA onset	Secondary ischemic injury	
**Disruption of neurovascular function**	SHR	Compromised neurovascular coupling in response to whisker stimulation	Primary hypertension	[67]
		Persistent vascular dysfunction after antihypertensive treatment		[71]
		↓ in the cortical hemodinamic response in six-months-old SHR		[18]
		Transition from early hyperperfusion to later hypoperfusion		[72]
	BPH	Reduced PVMs correlated with improved cognitive function	Primary hypertension	[67]

## Conclusions

Hypertension is a critical public health problem and, among cardiovascular diseases, stands as one of the most significant contributors in causing death. Hypertension is related to many adverse systemic effects, but the brain is among the first organs to be affected. In fact, by negatively acting on blood vessel structure and function, hypertension can correlate with stroke, cognitive impairment related to vascular causes, and Alzheimer’s disease. As mentioned, in reaction to increased pressure, remodeling and stiffening occur at the vessel level which can decrease cerebrovascular reserve. It is well known that a proper supply of oxygen and glucose is necessary for the brain. In the presence of deregulated blood pressure, this supply is compromised because autoregulation cannot be ensured. Lack of autoregulation can lead to several consequences including BBB dysfunction, an early indicator of Alzheimer’s disease. In addition, a prolonged increase in blood pressure due to angiotensin II results in the remodeling of tight junctions leading to permeability dysfunction. Interestingly, ROS plays a key role in brain damage. Produced mainly by PVMs, ROS contributes largely to neurovascular dysfunction. Having appropriate animal models is a crucial factor for the study of diseases even though there are intrinsic limitations. Rodent models, particularly SHR, have given significant intuitions into the mechanisms of hypertension-induced brain pathology. These models have demonstrated that hypertension leads to vascular remodeling, microcirculatory dysfunction, and BBB disruption. The accumulation of extracellular matrix, thickening of capillary walls, and damage to pericytes are key features observed in hypertensive brains. Additionally, the function of angiotensin II (Ang II) in promoting vascular changes and BBB permeability has been highlighted. These findings underscore the significance of timely identification and management of high blood pressure to prevent long-term neurological consequences.

Although much progress has been made in research, there are still several gaps in understanding the triggers, molecular mechanisms underlying pathogenesis, interaction between arterial abnormality and tissue damage, potential reversibility, pharmacological targets, and molecular biomarkers. Understanding these additional aspects is crucial to developing effective and efficient therapies. Future research should prioritize various critical areas to further elucidate the relationship between hypertension and brain damage. Specifically, understanding the precise pathways involved can facilitate the advancement of targeted therapies. Therapeutic interventions should be explored to assess the efficacy of novel antihypertensive treatments and their potential to mitigate cerebrovascular damage.

Answering these and other questions is a great stimulus for research in this field: it is imperative to develop better therapeutic approaches than the current ones, as well as to define innovative biological and molecular markers that can help in the early identification of hypertension-related brain damage.

## Data Availability

This article is based on a review of existing literature. Data supporting the findings are available from the corresponding author upon reasonable request.

## Abbreviations

BP: Blood Pressure
BBB: Blood-brain barrier
BPH/2J: Blood Pressure High 2 J
SHR: Spontaneously Hypertensive Rats
SHR-SP: Spontaneously Hypertensive Rats Stroke Prone
CSVD: Cerebral small vessel disease
CBF: Cerebral blood flow
ROS: Reactive oxygen species
PVMs: Perivascular macrophages
WKY: Wistar Kyoto rats
TJ: Tight junctions
VCI: Vascular cognitive impairment

## References

[1] Iqbal AM, Jamal SF. Essential Hypertension. 2023 Jul 20. In: StatPearls. Treasure Island (FL): StatPearls Publishing. 2026 Jan. PMID: 30969681. 30969681

[2] Franco C, Sciatti E, Favero G, Bonomini F, Vizzardi E, Rezzani R. Essential Hypertension and Oxidative Stress: Novel Future Perspectives. IJMS. 2022;23(22):14489. 36430967 10.3390/ijms232214489 PMC9692622

[3] Kelly DM, Rothwell PM. Blood pressure and the brain: the neurology of hypertension. Pract Neurol. 2020;20(2):100–8. 31558584 10.1136/practneurol-2019-002269

[4] Santisteban MM, Ahn SJ, Lane D, Faraco G, Garcia-Bonilla L, Racchumi G, et al. Endothelium Macrophage Crosstalk Mediates Blood-Brain Barrier Dysfunction in Hypertension. Hypertension. 2020;76(3):795–807. 32654560 10.1161/HYPERTENSIONAHA.120.15581 PMC7429290

[5] Santisteban MM, Iadecola C, Carnevale D. Hypertension, Neurovascular Dysfunction, and Cognitive Impairment. Hypertension. 2023;80(1):22–34. 36129176 10.1161/HYPERTENSIONAHA.122.18085 PMC9742151

[6] Özkan E, Çetin-Taş Y, Şekerdağ E, Kızılırmak AB, Taş A, Yıldız E, et al. Blood–brain barrier leakage and perivascular collagen accumulation precede microvessel rarefaction and memory impairment in a chronic hypertension animal model. Metab Brain Dis. 2021;36(8):2553–66. 34118020 10.1007/s11011-021-00767-8

[7] Hainsworth AH, Markus HS, Schneider JA. Cerebral Small Vessel Disease, Hypertension, and Vascular Contributions to Cognitive Impairment and Dementia. Hypertension. 2024;81(1):75–86. 38044814 10.1161/HYPERTENSIONAHA.123.19943 PMC10734789

[8] Liu N, Li N, Cao X, et al. More severe vascular remodeling in deep brain regions caused by hemodynamic differences is a potential mechanism of hypertensive cerebral small vessel disease. J Cereb Blood Flow Metab. 2025;45(8):1593–605. 40119683 10.1177/0271678X251327919 PMC11948236

[9] Bastrup JA, Jepps TA. Proteomic mapping reveals dysregulated angiogenesis in the cerebral arteries of rats with early-onset hypertension. J Biol Chem. 2023;299(10):105221. 37660920 10.1016/j.jbc.2023.105221 PMC10558802

[10] Suri S, Chiesa ST, Zsoldos E, Mackay CE, Filippini N, Griffanti L, et al. Associations between arterial stiffening and brain structure, perfusion, and cognition in the Whitehall II Imaging Sub-study: A retrospective cohort study. Willey JZ, editor. PLoS Med. 2020;17(12):e1003467. 33373359 10.1371/journal.pmed.1003467 PMC7771705

[11] Coverdale NS, Champagne AA, Allen MD, et al. Brain atrophy, reduced cerebral perfusion, arterial stiffening, and wall thickening with aging coincide with stimulus-specific changes in fMRI-BOLD responses. Am J Physiol-Regul Integr Comp Physiol. 2024;326(5):R346–56. 38406844 10.1152/ajpregu.00270.2023

[12] Shekhar S, Liu R, Travis OK, Roman RJ, Fan F. Cerebral Autoregulation in Hypertension and Ischemic Stroke: A Mini Review. J Pharm Sci Exp Pharmacol. 2017;2017(1):21–7. 29333537 PMC5765762

[13] Supti ST, Koehn LM, Newman SA, Pan Y, Nicolazzo JA. Iron Reduces the Trafficking of Fatty Acids from Human Immortalised Brain Microvascular Endothelial Cells Through Modulation of Fatty Acid Transport Protein 1 (FATP1/SLC27A1). Pharm Res. 2024;41(8):1631–48. 39044044 10.1007/s11095-024-03743-w PMC11362236

[14] Huang J, Oh M, Robert C, Huang X, Egle M, Tozer DJ, et al. Loss of white matter integrity mediates the association between cortical cerebral microinfarcts and cognitive dysfunction: A longitudinal study. J Cereb Blood Flow Metab. 2024;44(10):1723–32. 38796858 10.1177/0271678X241258563 PMC11494832

[15] Jandke S, Garz C, Schwanke D, et al. The association between hypertensive arteriopathy and cerebral amyloid angiopathy in spontaneously hypertensive stroke-prone rats. Brain Pathol. 2018;28(6):844–59. 30062722 10.1111/bpa.12629 PMC8028507

[16] Wenzel P, Knorr M, Kossmann S, Stratmann J, Hausding M, Schuhmacher S, et al. Lysozyme M– Positive Monocytes Mediate Angiotensin II–Induced Arterial Hypertension and Vascular Dysfunction. Circulation. 2011;124(12):1370–81. 21875910 10.1161/CIRCULATIONAHA.111.034470

[17] Naessens DMP, De Vos J, Richard E, Wilhelmus MMM, Jongenelen CAM, Scholl ER, et al. Effect of 518 long-term antihypertensive treatment on cerebrovascular structure and function in hypertensive rats. Sci Rep. 2023;13(1):3481. 36859481 10.1038/s41598-023-30515-0 PMC9977931

[18] Crofts A, Trotman-Lucas M, Janus J, et al. Longitudinal Multimodal fMRI to Investigate Neurovascular Changes in Spontaneously Hypertensive Rats. J Neuroimaging. 2020;30(5):609–16. 32648648 10.1111/jon.12753

[19] Hung T-H, Chen VC-H, Chuang Y-C, et al. Investigating the effect of hypertension on vascular cognitive impairment by using the resting-state functional connectome. Sci Rep. 2024;14(1):4580. 38403657 10.1038/s41598-024-54996-9 PMC10894879

[20] Lerman LO, Kurtz TW, Touyz RM, et al. Animal Models of Hypertension: A Scientific Statement From the American Heart Association. Hypertension. 2019;73(6):e87–120. 30866654 10.1161/HYP.0000000000000090 PMC6740245

[21] Takeuchi F, Liang Y-Q, Isono M, et al. Integrative genomic analysis of blood pressure and related phenotypes in rats. Dis Model Mech. 2021;14(5):dmm048090. 34010951 10.1242/dmm.048090 PMC8188887

[22] Ueno M, Chiba Y, Matsumoto K, Murakami R, Fujihara R, Kawauchi M, et al. Blood-brain barrier damage in vascular dementia. Neuropathology. 2016;36(2):115–24. 26607405 10.1111/neup.12262

[23] Okamoto K, Yamamoto K, Morita N, Ohta Y, Chikugo T, Higashizawa T, et al. Establishment and use 461 of the M strain of stroke-prone spontaneously hypertensive rat. J Hypertens Suppl. 1986;4(3):S21–4. 3465899

[24] Yamori Y, Sagara M, Mori H, Mori M. Stroke-Prone SHR as Experimental Models for Cardiovascular 464 Disease Risk Reduction in Humans. Biomedicines. 2022;10(11):2974. 36428542 10.3390/biomedicines10112974 PMC9687971

[25] Hannawi Y, Caceres E, Ewees MG, et al. Characterizing the Neuroimaging and Histopathological Correlates of Cerebral Small Vessel Disease in Spontaneously Hypertensive Stroke-Prone Rats. Front Neurol. 2021;12:740298. 34917012 10.3389/fneur.2021.740298 PMC8669961

[26] Lee M, Leskova W, Eshaq RS, Amezquita Z, Harris NR. Mechanisms of retinal photoreceptor loss in 470 spontaneously hypertensive rats. Exp Eye Res. 2024;247:110065. 39222765 10.1016/j.exer.2024.110065 PMC11412233

[27] Van Ham WB, Kessler EL, Oerlemans MIFJ, Handoko ML, Sluijter JPG, Van Veen TAB, et al. Clinical 472 Phenotypes of Heart Failure With Preserved Ejection Fraction to Select Preclinical Animal Models. 473 JACC. Basic Trans Sci. 2022;7(8):844–57. 10.1016/j.jacbts.2021.12.009 PMC9436760 36061340

[28] Basuli D, Parekh RU, White A, et al. Kinin B1 Receptor Mediates Renal Injury and Remodeling in Hypertension. Front Med (Lausanne). 2022;8:780834. 35118089 10.3389/fmed.2021.780834 PMC8804098

[29] Ferrari LF, Rey C, Ramirez A, et al. Characterization of the Dahl salt-sensitive rat as a rodent model of inherited, widespread, persistent pain. Sci Rep. 2022;12(1):19348. 36369350 10.1038/s41598-022-24094-9 PMC9652451

[30] Mullins L, Ivy J, Ward M, Tenstad O, Wiig H, Kitada K, et al. Abnormal neonatal sodium handling in 481 skin precedes hypertension in the SAME rat. Pflugers Arch. 2021;473(6):897–910. 34028587 10.1007/s00424-021-02582-7 PMC8164623

[31] Jackson KL, Head GA, Gueguen C, et al. Mechanisms Responsible for Genetic Hypertension in Schlager BPH/2 Mice. Front Physiol. 2019;10:1311. 31681017 10.3389/fphys.2019.01311 PMC6813185

[32] Schlager G. Selection for blood pressure levels in mice. Genetics. 1974;76(3):537–49. 4833575 10.1093/genetics/76.3.537 PMC1213083

[33] Mouat MA, Jackson KL, Coleman JLJ, et al. Deletion of Orphan G Protein-Coupled Receptor GPR37L1 in Mice Alters Cardiovascular Homeostasis in a Sex-Specific Manner. Front Pharmacol. 2021;11:600266. 33633567 10.3389/fphar.2020.600266 PMC7901490

[34] Potje SR, Isbatan A, Tostes RC, Bendhack LM, Dull RO, Carvalho-de-Souza JL, et al. Glypican 1 and 492 syndecan 1 differently regulate noradrenergic hypertension development: Focus on IP3R and calcium. 493 Pharmacol Res. 2021;172:105813. 34411733 10.1016/j.phrs.2021.105813 PMC10200078

[35] Daghbouche-Rubio N, Álvarez-Miguel I, Flores VA, et al. The P2Y6 Receptor as a Potential Keystone in Essential Hypertension. Function (Oxf). 2024;5(6):zqae045. 39322240 10.1093/function/zqae045 PMC11577605

[36] Marques FZ, Campain AE, Davern PJ, Yang YHJ, Head GA, Morris BJ. Global identification of the genes and pathways differentially expressed in hypothalamus in early and established neurogenic 500 hypertension. Physiol Genomics. 2011;43(12):766–71. 21487032 10.1152/physiolgenomics.00009.2011

[37] Guillaumin MCC, Burdakov D. Neuropeptides as Primary Mediators of Brain Circuit Connectivity. Front Neurosci. 2021;15:644313. 33776641 10.3389/fnins.2021.644313 PMC7991401

[38] Guyenet PG, Stornetta RL, Souza GMPR, Abbott SBG, Brooks VL. Neuronal Networks in 505 Hypertension: Recent Advances. Hypertension. 2020;76(2):300–11. 32594802 10.1161/HYPERTENSIONAHA.120.14521 PMC7347452

[39] Jackson KL, Dampney BW, Moretti JL, Stevenson ER, Davern PJ, Carrive P, et al. Contribution of Orexin to the Neurogenic Hypertension in BPH/2J Mice. Hypertension. 2016;67(5):959–69. 26975709 10.1161/HYPERTENSIONAHA.115.07053

[40] Improta-Caria AC, Aras MG, Nascimento L, De Sousa RAL, Aras-Júnior R, Souza BSDF. MicroRNAs Regulating Renin–Angiotensin–Aldosterone System, Sympathetic Nervous System and Left Ventricular Hypertrophy in Systemic Arterial Hypertension. Biomolecules. 2021;11(12):1771. 34944415 10.3390/biom11121771 PMC8698399

[41] Jäkel L, De Kort AM, Klijn CJM, et al. Prevalence of cerebral amyloid angiopathy: A systematic review and meta-analysis. Alzheimers Dement. 2022;18(1):10. 513 28. 34057813 10.1002/alz.12366 PMC9290643

[42] Sieminski M, Szypenbejl J, Partinen E. Orexins, Sleep, and Blood Pressure. Curr Hypertens Rep. 2018;20(9):79. 29992504 10.1007/s11906-018-0879-6 PMC6061179

[43] Pires PW, Dams Ramos CM, Matin N, et al. The effects of hypertension on the cerebral circulation. Am J Physiol-Heart Circ Physiol. 2013;304(12):H1598–614. 23585139 10.1152/ajpheart.00490.2012 PMC4280158

[44] Chillon JM, Baumbach GL. Effects of an Angiotensin-Converting Enzyme Inhibitor and a β-Blocker on Cerebral Arterioles in Rats. Hypertension. 1999;33(3):856–61. 10082499 10.1161/01.hyp.33.3.856

[45] Sabharwal R, Chapleau MW, Gerhold TD, Baumbach GL, Faraci FM. Plasticity of cerebral microvascular structure and mechanics during hypertension and following recovery of arterial pressure. Am J Physiol Heart Circ Physiol. 2022;323(6):H1108–17. 36269650 10.1152/ajpheart.00292.2022 PMC9678426

[46] Wang Q, Lao M, Xu Z, et al. Caveolin–1 modulates hypertensive vascular remodeling via regulation of the Notch pathway. Mol Med Rep. 2020;22(5):4320–8. 33000233 10.3892/mmr.2020.11508 PMC7533525

[47] Cipolla MJ, Chan SL. Impact of Acute and Chronic Hypertension on Changes in Pial Collateral Tone In Vivo During Transient Ischemia. Hypertension. 2020;76(3):1019–26. 32683904 10.1161/HYPERTENSIONAHA.120.15356 PMC7429292

[48] Aimagambetova B, Ariko T, Merritt S, Rundek T. Arterial stiffness measured by pulse wave velocity correlated with cognitive decline in hypertensive individuals: a systematic review. BMC Neurol. 2024;24(1):393. 39415095 10.1186/s12883-024-03905-8 PMC11481605

[49] Ajamu SO, Fenner RC, Grigorova YN, Cezayirli D, Morrell CH, Lakatta EG, et al. Association of central arterial stiffness with hippocampal blood flow and N-acetyl aspartate concentration in hypertensive adult Dahl salt sensitive rats. J Hypertens. 2021;39(10):2113–21. 34001812 10.1097/HJH.0000000000002899 PMC8452328

[50] Owens WB. Blood pressure control in acute cerebrovascular disease. J Clin Hypertens (Greenwich). 2011;13(3):205–11. 21366852 10.1111/j.1751-7176.2010.00394.x PMC8673094

[51] Cipolla MJ, Liebeskind DS, Chan SL. The importance of comorbidities in ischemic stroke: Impact of hypertension on the cerebral circulation. J Cereb Blood Flow Metab. 2018;38(12):2129–49. 30198826 10.1177/0271678X18800589 PMC6282213

[52] Sweeney MD, Zhao Z, Montagne A, et al. Blood-Brain Barrier: From Physiology to Disease and Back. Physiol Rev. 2019;99(1):21–78. 30280653 10.1152/physrev.00050.2017 PMC6335099

[53] Huang B, Li X. The Role of Mfsd2a in Nervous System Diseases. Front Neurosci. 2021;15:730534. 34566571 10.3389/fnins.2021.730534 PMC8461068

[54] Divecha YA, Rampes S, Tromp S, Boyanova ST, Fleckney A, Fidanboylu M, et al. The microcirculation, the blood-brain barrier, and the neurovascular unit in health and Alzheimer disease: The aberrant pericyte is a central player. Pharmacol Rev. 2025;77(3):100052. 40215558 10.1016/j.pharmr.2025.100052 PMC12163501

[55] Lai AY, Joo IL, Trivedi AU, Dorr A, Hill ME, Stefanovic B, et al. Cerebrovascular damage after midlife transient hypertension in non-transgenic and Alzheimer’s disease rats. Brain Res. 2021;1758:147369. 33582120 10.1016/j.brainres.2021.147369 PMC8005286

[56] Guo S, Som AT, Arai K, Lo EH. Effects of angiotensin-II on brain endothelial cell permeability via PPARalpha regulation of para- and trans-cellular pathways. Brain Res. 2019;1722:146353. 31356784 10.1016/j.brainres.2019.146353 PMC6755037

[57] Ueno M, Sakamoto H, Tomimoto H, Akiguchi I, Onodera M, Huang CL, et al. Blood-brain barrier is impaired in the hippocampus of young adult spontaneously hypertensive rats. Acta Neuropathol. 2004;107(6):532–8. 15042385 10.1007/s00401-004-0845-z

[58] Iadecola C, Duering M, Hachinski V, Joutel A, Pendlebury ST, Schneider JA, et al. Vascular Cognitive Impairment and Dementia. J Am Coll Cardiol. 2019;73(25):3326–44. 31248555 10.1016/j.jacc.2019.04.034 PMC6719789

[59] Mustapha M, Nassir CMNCM, Aminuddin N, et al. Cerebral Small Vessel Disease (CSVD) – Lessons From the Animal Models. Front Physiol. 2019;10:1317. 31708793 10.3389/fphys.2019.01317 PMC6822570

[60] Inoue Y, Shue F, Bu G, et al. Pathophysiology and probable etiology of cerebral small vessel disease in vascular dementia and Alzheimer’s disease. Mol Neurodegener. 2023;18(1):46. 37434208 10.1186/s13024-023-00640-5 PMC10334598

[61] Tarkkonen A, Kyläheiko I, Inkeri J, et al. Progression of Cerebral Small Vessel Disease Among Neurologically Asymptomatic Middle-Aged Individuals With Type 1 Diabetes. Diabetes Care. 2025;48(5):776–80. 40095501 10.2337/dc24-1825

[62] Broderick M, Rosignoli L, Lunagariya A, et al. Hypertension is a Leading Cause of Nontraumatic Intracerebral Hemorrhage in Young Adults. J Stroke Cerebrovasc Dis. 2020;29(5):104719. 32122779 10.1016/j.jstrokecerebrovasdis.2020.104719 PMC7188522

[63] Carlsson R, Enström A, Paul G. Molecular Regulation of the Response of Brain Pericytes to Hypoxia. Int J Mol Sci. 2023;24(6):5671. 36982744 10.3390/ijms24065671 PMC10053233

[64] Yang X, Wang J, Zhang J, et al. Cerebrovascular-mediated dynamic alterations in neurovascular coupling: a key pathological mechanism of depression. Cell Biosci. 2025;15(1):97. 40624522 10.1186/s13578-025-01444-4 PMC12232600

[65] Dabertrand F, Harraz OF, Koide M, Longden TA, Rosehart AC, Hill-Eubanks DC, et al. PIP 2 corrects cerebral blood flow deficits in small vessel disease by rescuing capillary Kir2.1 activity. Proc Natl Acad Sci USA. 2021;118(17):e2025998118. 33875602 10.1073/pnas.2025998118 PMC8092380

[66] Iadecola C, Gottesman RF. Neurovascular and Cognitive Dysfunction in Hypertension: Epidemiology, Pathobiology, and Treatment. Circ Res. 2019;124(7):1025–44. 30920929 10.1161/CIRCRESAHA.118.313260 PMC6527115

[67] Faraco G, Sugiyama Y, Lane D, et al. Perivascular macrophages mediate the neurovascular and cognitive dysfunction associated with hypertension. J Clin Invest. 2016;126(12):4674–89. 27841763 10.1172/JCI86950 PMC5127678

[68] Capone C, Faraco G, Peterson JR, Coleman C, Anrather J, Milner TA, et al. Central cardiovascular circuits contribute to the neurovascular dysfunction in angiotensin II hypertension. J Neurosci. 2012;32(14):4878–86. 22492044 10.1523/JNEUROSCI.6262-11.2012 PMC3328774

[69] Santisteban MM, Iadecola C. Hypertension, dietary salt and cognitive impairment. J Cereb Blood Flow Metab. 2018;38(12):2112–28. 30295560 10.1177/0271678X18803374 PMC6282225

[70] Kazama K, Anrather J, Zhou P, Girouard H, Frys K, Milner TA, et al. Angiotensin II Impairs Neurovascular Coupling in Neocortex Through NADPH Oxidase–Derived Radicals. Circ Res. 2004;95(10):1019–26. 15499027 10.1161/01.RES.0000148637.85595.c5

[71] Calcinaghi N, Wyss MT, Jolivet R, Singh A, Keller AL, Winnik S, et al. Multimodal Imaging in Rats Reveals Impaired Neurovascular Coupling in Sustained Hypertension. Stroke. 2013;44(7):1957–64. 23735955 10.1161/STROKEAHA.111.000185

[72] Li Y, Li R, Liu M, et al. MRI study of cerebral blood flow, vascular reactivity, and vascular coupling in systemic hypertension. Brain Res. 2021;1753:147224. 33358732 10.1016/j.brainres.2020.147224

